# Consumer involvement in Quality Use of Medicines (QUM) projects – lessons from Australia

**DOI:** 10.1186/1472-6963-5-75

**Published:** 2005-12-01

**Authors:** Carl MJ Kirkpatrick, Elizabeth E Roughead, Gregory R Monteith, Susan E Tett

**Affiliations:** 1 School of Pharmacy, University of Queensland, Brisbane, Australia; 2 Quality Use of Medicines and Pharmacy Research Centre, School of Pharmacy and Medical Sciences, University of South Australia, Adelaide, Australia

## Abstract

**Background:**

It is essential that knowledge gained through health services research is collated and made available for evaluation, for policy purposes and to enable collaboration between people working in similar areas (capacity building). The Australian Quality Use of Medicine (QUM) on-line, web-based project database, known as the QUMmap, was designed to meet these needs for a specific sub-section of health services research related to improving the use of medicines. Australia's National Strategy for Quality Use of Medicines identifies the primacy of consumers as a major principle for quality use of medicines, and aims to support consumer led research. The aim of this study was to determine how consumers as a group have been represented in QUM projects in Australia. A secondary aim was to investigate how the projects with consumer involvement fit into Australia's QUM policy framework.

**Method:**

Using the web-based QUMmap, all projects which claimed consumer involvement were identified and stratified into four categories, projects undertaken by; (a) consumers for consumers, (b) health professionals for consumers, (c) health professionals for health professionals, and (d) other. Projects in the first two categories were then classified according to the policy 'building blocks' considered necessary to achieve QUM.

**Results:**

Of the 143 'consumer' projects identified, the majority stated to be 'for consumers' were either actually by health professionals for health professionals (c) or by health professionals for consumers (b) (47% and 40% respectively). Only 12 projects (9%) were directly undertaken by consumers or consumer groups for consumers (a). The majority of the health professionals for consumers (b) projects were directed at the provision of services and interventions, but were not focusing on the education, training or skill development of consumers.

**Conclusion:**

Health services research relating to QUM is active in Australia and the projects are collated and searchable on the web-based interactive QUMmap. Healthcare professionals appear to be dominating nominally 'consumer focussed' research, with less than half of these projects actively involving the consumers or directly benefiting consumers. The QUMmap provides a valuable tool for policy analysis and for provision of future directions through identification of QUM initiatives.

## Background

Many of the findings of health services research are never published in peer-reviewed journals nor presented at a conference [1,2]. There may be many reasons for this, including that such projects are often small, the researchers often hold clinical or service positions and their research time is minimal or non-existent, or the results did not eventuate 'as intended' [1]. A lot of potential 'lessons learned' are lost to future researchers, there is a risk that an unsuccessful study may be replicated, the researchers themselves lose an opportunity to make contact with other researchers with similar interests or similar studies, and input into policy direction or development is lost [2]. Opportunities for capacity building by teams linking up together are also lost. As discussed below, the quality use of medicines (QUM) on-line project database, known as the QUMmap [3], seeks to address some of these issues and provide decision makers with an interactive, accessible web-based database to search for previous and on-going QUM initiatives.

Initiatives to improve use of medicines are being implemented around the world in an effort to maximise health outcomes, reduce adverse events and keep health costs within affordable limits. While prescribing behaviour is one factor that can be targeted to improve use of medicines, it is widely acknowledged that consumer behaviour also influences medication use and that involvement of consumers in strategies to improve use of medicines is necessary in any country's attempts to promote rational drug use [4].

Australia has a comprehensive strategy for promoting rational drug use, known as the National Strategy for QUM [5]. A key principle of the strategy is the primacy of consumers in any initiative to promote quality use of medicines. Another key principle is that multidisciplinary, collaborative approaches are necessary to improve use of medicines and that these approaches should involve all stakeholder groups (ie. doctors, nurses, pharmacists, consumers etc) from the beginning of an initiative's development. This has demonstrated effectiveness particularly in the modification of drug use. [6,7] A WHO publication on developing and implementing national drug policies stated the importance of consumers in improving use of medicines [4].

To assist in evaluating the implementation of the National Strategy for Quality Use of Medicines, the QUM Mapping Project was commissioned by the Australian Department of Health and Ageing, in conjunction with the Pharmaceutical Health and Rational use of Medicines (PHARM) committee [2]. The aim was to set up a database of QUM projects and initiatives in Australia. The mapping project website has been on-line since February 1999, and has collected over 1000 reports of QUM projects in Australia.

**Figure 1:**
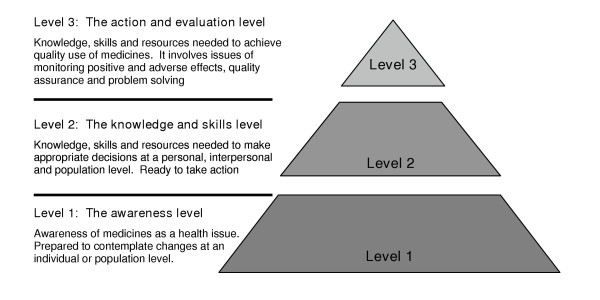
Levels of the QUM pyramid – the faces of the three dimensional pyramid representing all the partners required to achieve optimal quality use of medicines (consumers, government, health professionals, industry). Adapted from the National Strategy for Quality Use of Medicines – Plain English Edition [8]

The approach to improving medicine use in Australia is often referred to as a systems approach, indicating that behaviours to support quality use of medicines must be developed, while at the same time a supportive environment must be created. In developing appropriate behaviours, there is a need to implement initiatives that raise awareness of quality use of medicines as an issue, that develop appropriate skills and knowledge, and that reinforce and maintain appropriate behaviours [8]. This is conceptualised as Figure 1. The National Strategy identifies six 'building blocks' as essential components to achieve optimal quality of medicine use, raising levels of awareness and action across all sectors;

• Policy development and implementation;

• Facilitation and co-ordination of quality use of medicines initiatives;

• Provision of objective information and assurance of ethical promotion of medicines;

• Education and training;

• Provision of services and appropriate interventions; and

• Strategic research, evaluation and routine data collection.

This strategy also has international relevance, as the health care systems of many countries strive to improve the use of medications and to increase consumer involvement in this process [9-11].

The consumer movement in Australia was a driving force behind the establishment of the National Strategy for Quality Use of Medicines and Australia's National Medicines Policy. In 1988, the Consumers Health Forum of Australia circulated a discussion paper, entitled "Developing a rational medicinal drug policy for Australia – What does it mean?" [12]. This document was published in its newsletter and distributed to Commonwealth, State and Territory governments, and to the organisations which represented pharmacists, medical practitioners, the pharmaceutical industry, clinical pharmacologists, alcohol and drug societies and patient support groups. In 1989, the Consumers Health Forum of Australia moved the process forward again when they produced a report entitled "Towards a National Medicinal Drug Policy" [13]. This identified a number of components considered, from a consumer perspective, to be essential within a national medicines policy. The lack of any overall policy on quality medication use was noted. In addition, maintaining services developed by and for consumers was seen as an important component of any national strategy. The implementation of Australia's National Strategy for Quality Use of Medicines, which began in 1992, was supported with approximately $2 million annually in research funding. This research funding was available to all groups and aimed to ensure multidisciplinary research, which included consumer involvement from project conceptualisation. There were also efforts to support consumer led research so that services and resources appropriate to consumer needs for improving use of medicines were developed. More recently, Australia's National Health and Medical Research Council have published a statement on consumer and community participation in health and medical research [14]. Consumer involvement in research has also been investigated using surveys and questionnaires to determine the nature and extent of their involvement in funding bodies and in randomised clinical trials [15,16].

### Objectives

The main aim of this study was to evaluate how consumers, as a group, have been represented in the QUM projects in Australia. A secondary aim was to investigate how these projects relate to the QUM policy framework, in particular, to the six key QUM building blocks defined by Australia's National Strategy for QUM.

## Methods

Using the standard SQL search engine available on the QUMmap [3], all projects were identified which either claimed that "consumers" were the target group (this is a tick box available as one choice when projects are entered on the database) or included a text word "consumer". The term "consumer" is widely used in reference to healthcare in Australia and has a broad definition to include any user or potential user of health care [17]. The QUMmap has this group noted as a specific tick box target group. The search was undertaken to be as inclusive as possible of all research involving consumers in any way, for consultation, for collaboration or as initiators of the research. The database search was undertaken for the period February 1999 and December 2004 using the publicly accessible searching facilities provided on the website [3].

All identified projects were evaluated and sorted into four groups by CK, in consultation with ER and ST, using definitions as follows:

(a) Consumers for consumers – any consumer or consumer group undertaking research/initiatives directly for consumers;

(b) Health professionals for consumers – health professionals (doctors, nurses, pharmacists etc) undertaking research/initiatives which directly involve consumers as part of the initiative (eg health professionals working with consumer groups to provide educational material);

(c) Health professionals for health professionals – a QUM research project/initiative undertaken by health professionals to improve health professional services which may indirectly provide assistance to the consumer.

(d) Other – does not fit into any of these categories, although consumers had been nominated as a target group or the term consumer was included in the project description.

Categories (c) and (d) were discarded from further analysis because they did not involve consumers directly in QUM activities or research. This left the consumer for consumer for consumer (n = 12) (category (a)) and health professional for consumer (n = 69) (category (b)) projects (n = 81, total). These remaining projects were then allocated by CK, in consultation with ER and ST, to one of the three levels of the QUM pyramid (Figure 1) [8] and analysed to see which level the projects were achieving in the QUM framework. They were also allocated to one of the six key building blocks of Australia's QUM strategy [8]. A Kruskal-Wallis test was used (difference considered significant if P < 0.05) to investigate differences between project costs in the categories, where these were recorded.

## Results

In the period from February 1999 to December 2004, 143 projects on the QUMmap [3] were identified by the search strategy. However, the majority of projects stated to be for consumers, were either undertaken by health professionals for health professionals (category (c)) or health professionals for consumers (category (b)). Only 12 projects could be identified as being directly undertaken by consumers or consumer groups specifically for consumers (category (a)) (Table 1).

**Table 1:** Categories of projects on the QUMmap identifying consumers as their target, or having consumer as a searchable term

Category	Number	Percent
Health professionals for consumers (b)	69	47
Health professionals for health professionals (c)	57	40
Consumers for consumers (a)	12	9
Other (d)	5	4

The geographical distribution of projects sorted by categories (a), (b) and (c) is presented in Table 2. The majority of projects in the consumers for consumers (a) category were undertaken in the state of New South Wales (8/12), Australia's most populous state where many head offices of consumer organisations are found. Five projects were undertaken by the Australian Pensioners' & Superannuants' Federation, one by the Council of the Aging, and one by the Combined Pensioners and Superannuants Association.

**Table 2:** State distribution of selected categories of project on the QUM Map

	Number (%)		
State	Consumers for consumers (a)	Health professionals for consumers (b)	Health professionals for health professionals (c)

NSW	8 (67)	17 (25)	18 (32)
SA	1 (8)	17 (25)	15 (26)
VIC	2 (17)	12 (17)	7 (12)
ACT	-	5 (7)	4 (7)
QLD	-	8 (12)	9 (16)
WA	-	7 (10)	3 (5)
TAS	-	2 (3)	1 (2)
NT	1 (8)	1 (1)	-

The median cost of the 9 consumers for consumers (category (a)) projects for which funding data were available was AUD $59,000 (range 26,000 – 240,000). This was similar to the median cost of $57,000 for health professionals for consumers (category (b)) projects (n = 45 with funding data available; range $400 – $668,000). The median cost for health professionals for health professionals (category (c)) projects was AUD $83,000 (n = 27 with funding data available, range $5,000 – $392,000). These were not statistically significantly different (P = 0.18).

The QUM pyramid levels for the health professionals for consumers (category (b)) and the consumers for consumers (category (a)) projects are presented in Table 3. Most of the consumers for consumers (category (a)) projects were about informing or educating consumers about QUM, with a small number of projects introducing skill levels to make appropriate QUM decisions. In comparison, the health professionals for consumers (category (b)) projects covered all levels of the QUM pyramid. It is interesting to note that more of the health professionals for consumers (category (b)) projects were located in the upper part of the pyramid, with a greater focus on action and evaluation.

**Table 3:** QUM pyramid level as defined in Australia's National Strategy for Quality Use of Medicines (QUM) for selected projects on the QUMmap

	QUM Pyramid level		
	Raising awareness	Knowledge and skill development	Reinforcement and maintenance

Consumers for consumers (a)	8	4	0
Health professionals for consumers (b)	24	20	25

Analysis of the projects according to the QUM building block classification demonstrated that the majority of health professionals for consumers (category (b)) projects were about the provision of services and interventions. The health professionals for consumers (category (b)) projects did not focus on education and training or building skill development in consumers.

## Discussion

This paper audits the consumer role in QUM projects listed on the Australian QUM Map, asking the question, are consumers adequately involved in the QUM activities in Australia? One hundred and forty three projects that claimed to involve "consumers" were identified using the searches readily available on the QUM map website [3]. Only 81 (57%) of these projects involved consumers either via consumer groups, or with some interaction with healthcare professionals to provide direct benefit to the consumer. Furthermore, only 12 (9%) were projects undertaken by consumers for consumers. The majority of the consumers for consumers projects have been undertaken in New South Wales, with Victoria being the next most prominent state. New South Wales and Victoria are Australia's most populated states.

A number of health professional projects (n = 69) are involving consumers (health professionals for consumers (category (b)) projects), with good distribution among all states of Australia. All projects that receive funding by the major supporting bodies for QUM in Australia are currently included on the QUMmap (more details of the projects included and the policy potential for the map have been published previously [2]). The data currently on the QUMmap can be considered to be a sample of projects undertaken in the QUM area in Australia, with the current audit showing a "snap-shot" of the spread of activity.

It would appear that health professionals for health professionals projects are receiving greater median funding per project than those directly involving consumers ($83,000 versus $57,000 median funding for health professionals for consumers or versus $59,000 median for consumers for consumers), although these differences were not statistically significant (P = 0.18, comparison of the three categories). Perhaps the level of expertise associated with grant applications by health professionals contrasted to those made by consumers or consumer groups. It could also be that project budgets are formulated differently, depending upon which partners are involved.

Evaluation using the levels of the QUM pyramid of the consumers for consumers (category (a)) and health professionals for consumers (category (b)) projects has shown some interesting trends. The consumers for consumers projects are mostly based at the bottom of the pyramid (information/awareness) with some in the middle sections endeavouring to increase awareness and prepare consumers to take action on QUM. In contrast, the health professionals for consumers projects are in the top two thirds of the pyramid – ready to take action and actively monitoring adverse and positive effects of QUM. In addition consumer projects, including those undertaken by health professionals are narrowly focused within the key building blocks, directed mainly to the provision of services and interventions. More activity across all the essential domains of Australia's QUM National Strategy is clearly required. Consumers (or consumer groups) need to be encouraged via financial, infrastructure or other means, to develop QUM projects that move into the upper echelon of the QUM pyramid and cover all key building blocks.

It is important that consumers are directly involved in all aspects of the QUM process in order to achieve successful behaviour change. It is well recognised that health professionals, by not directly involving consumers/consumer groups may be perceived as "paternalistic", the outcome being that consumers are resistant to being peripherally involved in activities or are resistant to changing their use of medicines [18-21]. Indeed, it has been shown that involvement of consumer and community groups early in projects improves uptake by consumers, and this has been demonstrated particularly in projects involving the use of sunscreens, cancer screening programs, smoking cessation programs, and in the treatment of asthma [21-26]. Moreover, we found no projects designed and implemented by consumers for health care professionals.

Although other countries do not have specific QUM strategies or even defined National Medicines Policies including QUM, the messages presented here for Australia can be taken and developed in other health care systems internationally. Increasing consumer led initiatives is important in all countries [15,16]. The projects audited by this study are available for international perusal on the QUMmap and the QUMmap, or a similar database would be a valuable addition to the health services research effort of other countries. Canada has recently developed a similar database [27].

The major limitation of this present audit is that the QUMmap may not capture all QUM research and projects implemented in Australia. However it does include all those that are funded by General Practice Education Programme (GPEP), QUM Evaluation Programme (QUMEP), National Health and Medical Research Council (NHMRC), Pharmacy Government Agreements, the National Prescribing Service (NPS) and the Safety and Quality Council. It has also achieved wide publicity such that many projects are entered by the investigators themselves [2]. Although this website has been advertised widely through academic, professional and other interested bodies, it is possible that some consumer based QUM projects have not been entered on to the database and therefore have not been included in this audit. The significance of the lack of mandatory reporting of QUM projects on the QUMmap has been highlighted previously [1]. Costings were included only for some projects, which limits the interpretation of the findings and statistical analyses of median funding for the different categories of projects. The projects were classified by CK, in consultation with ER and ST as consensus classifications. Although independent ratings and reliability analyses were not conducted, we consider that the classification system was clear and that misclassifications were unlikely.

## Conclusion

This audit of the QUMmap would indicate that consumers as a group are not well represented in QUM research in Australia, with some states having no consumer driven projects on the QUMmap. Healthcare professionals appear to be dominating consumer based research, with only a little over half of these projects actively involving the consumers or directly benefiting consumers. On this basis, consumers (or consumer groups) need to be encouraged via financial, infrastructure or other means, to develop or be directly involved in the QUM projects in all states of Australia. It is likely that similar conclusions could apply to other health care systems internationally.

## Competing interests

The author(s) declare that they have no competing interests.

## Authors' contributions

All four authors participated in the design of the study and assisted in data analysis and writing the manuscript. CK carried out the database searches and the collation of the results. ER and ST initially conceived the study.

## Pre-publication history

The pre-publication history for this paper can be accessed here:


